# Errata

**Published:** 1892-03

**Authors:** 


ERRATA.—Rev. T. M. McIntosh kindly calls our attention to the following typographical errors in his letters from London and Berlin:

In August (1891) issue, page 360, "Dr. Trachenix" should be "Dr. Trudeau"; page 361, "Mr. McEmon should be "Mr. McEwan." In November (1891) issue, page 553, top of page "urethra" should be "uterus." Second paragraph "slant" suture should be "stout" suture.

